# Microwave Spectroscopy Investigation of Carasau Bread Doughs: Effects of Composition up to 8.5 GHz

**DOI:** 10.3390/foods12122396

**Published:** 2023-06-16

**Authors:** Claudia Macciò, Andrea Melis, Matteo Bruno Lodi, Emanuele Garau, Francesco Desogus, Antonio Loddo, Fabrizio Di Napoli, Giuseppe Mazzarella, Alessandro Fanti

**Affiliations:** 1 Department of Electrical and Electronic Engineering, University of Cagliari, 09123 Cagliari, Italy; claudia.maccio9@gmail.com (C.M.); andrea.melis89@unica.it (A.M.); matteob.lodi@unica.it (M.B.L.); emanuelegarau92@gmail.com (E.G.); mazzarella@unica.it (G.M.); 2 Department of Mechanical, Chemical and Materials Engineering, University of Cagliari, 09123 Cagliari, Italy; francesco.desogus2@unica.it; 3 Il Vecchio Forno SUNALLE, Via Ogliastra, 10, 08023 Fonni, Italy; antonio.loddo@sunalle.it (A.L.); dinapolifabrizio@gmail.com (F.D.N.)

**Keywords:** Carasau bread, Cole–Cole model, dielectric permittivity, microwave spectroscopy, food characterization

## Abstract

Carasau bread is a flat bread, typical of Sardinia (Italy). The market of this food product has a large growth potential, and its industry is experiencing a revolution, characterized by digitalization and automation. To monitor the quality of this food product at different manufacturing stages, microwave sensors and devices could be a cost-effective solution. In this framework, knowledge of the microwave response of Carasau dough is required. Thus far, the analysis of the microwave response of Carasau doughs through dielectric spectroscopy has been limited to the dynamics of fermentation. In this work, we aim to perform complex dielectric permittivity measurements up to 8.5 GHz, investigating and modeling the role of water amount, salt and yeast concentrations on the spectra of this food product. A third-order Cole–Cole model was used to interpret the microwave response of the different samples, resulting in a maximum error of 1.58% and 1.60% for the real and imaginary parts of permittivity, respectively. Thermogravimetric analysis was also performed to support the microwave spectroscopy investigation. We found that dielectric properties of Carasau bread doughs strongly depend on the water content. The analysis highlighted that an increase in water quantity tends to increase the bounded water fraction at the expense of the free water fraction. In particular, the free water amount in the dough is not related to the broadening parameter $\gamma_{2}$ of the second pole, whereas the bound water weight fraction is more evident in the $\gamma_{2}$ and $\sigma_{dc}$ parameters. An increase in electrical conductivity was observed for increasing water content. The microwave spectrum of the real part of the complex permittivity is slightly affected by composition, while large variation in the imaginary part of the complex dielectric permittivity can be identified, especially for frequencies below 4 GHz. The methodology and data proposed and reported in this work can be used to design a microwave sensor for retrieving the composition of Carasau bread doughs through their dielectric signature.

## 1. Introduction

**Figure 1:**
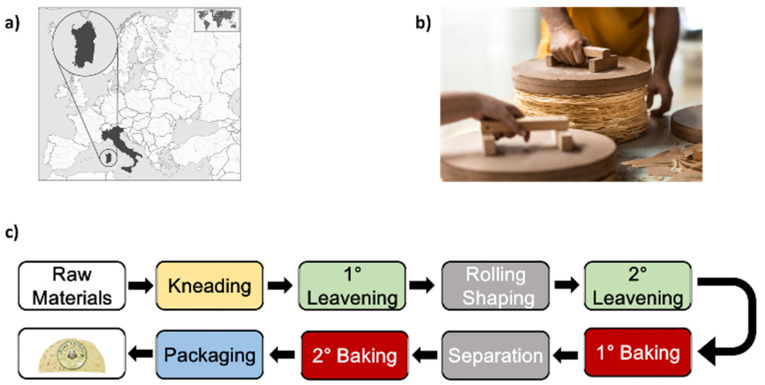
(**a**) Geographical location of Sardinia in the Mediterranean Sea. (**b**) Photo of Carasau bread (courtesy of SUNALLE). (**c**) Production process of Carasau bread.

Carasau bread (CB) or “pane carasau” is a typical and traditional food product from the island of Sardinia, Italy (Figure 1a) [1]. CB can be classified as a flat bread (FB), sharing several features with similar food products from the Mediterranean area [1,2]. CB is a circular bread, with a diameter ranging from 18 to 40 cm (Figure 1b), with a unique crisp texture and taste [3]. It is made of re-milled durum wheat semolina that is mixed with de-ionized water, iodized salt (NaCl) and baker’s yeast (*Saccharomyces cerevisiae*) [4]. As shown in Figure 1c, the ingredients are mixed for 20 min in a kneading machine to obtain the dough, which ferments for 30 min, and it is then sheeted into disks to undergo a second, longer, leavening in a dedicated room, for 40 min. After that, a first baking step is carried out at temperatures of ~570 °C. The dough disks dry and inflate. When the disks cool down, they are manually separated, and the crusts are baked again at ~400 °C for ~20 s. CB is packed in plastic films, then labeled for distribution and sold. Its market has large potential growth for several reasons. CB is a sustainable product since it does not need tableware, leading to a small water consumption, while being cooked in very short times [1]. Also, being a local product, it is not necessary to import raw materials to carry on its production. Furthermore, since it has been demonstrated that Sardinian dietary habits are strongly correlated with longevity (this Italian region hosts one of the world’s blue zones), Carasau is attractive to consumers [5]. Indeed, foreign cuisines, such as Danish [6], are also adopting and discovering this Sardinian flat bread. In this framework, the CB industry has been demonstrating the will to change and improve to follow this trend. Since 2007, the problem of the automation of CB production process was faced [7], while engineering studies were carried out to design advanced bakery plants [8]. At present, Carasau bakeries are experiencing a revolution. The modeling of the CB production process through the use of hybrid Petri nets was recently reported [9]. A wireless sensor network (WSN) was designed, developed and tested to study the most relevant processing (e.g., conveyor belt velocities) and environmental parameters (e.g., ambient temperature, air pressure, gas concentration, etc.) [10]. However, these engineering tools are often not enough to empower and support the development of the production process. In particular, as recently underlined in recent studies [11,12,13], the acquired process and environmental parameters must be combined, analyzed and interpreted in a complex multifactorial quantitative framework for evaluating the evolution of the physical properties of the semi-processed elements or the quality of the final products.

In this framework, it is considered necessary to expand the knowledge about the physical properties of Carasau bread doughs. From this extended understanding, new devices and tools could be devised, designed and used. As regards the CB, some physical and chemical characterization methods have been used to assess the fundamental properties and quality features of the doughs. In particular, rheological properties of Carasau doughs have been investigated considering different wheat varieties, various processing conditions (i.e., mixing times, stirring conditions, etc.) and the influence of composition [14,15]. Thermogravimetric and calorimetric features of Carasau doughs have also been investigated, considering variable water, yeast and salt contents [16]. Recently, low-field nuclear magnetic resonance (NMR) was employed to investigate how water and flour percentages influence the dough microstructure [17]. In ref. [18], indirect Fourier transform infrared (FTIR) characterization was performed on Carasau dough and correlated to the rheological parameters to establish a strong modeling of the dough features. A similar approach was undertaken in ref. [19], where cryogenic dielectric spectroscopy measurements were performed from 0.1 Hz up to 10 MHz, and these data were correlated with rheological quantities.

Although all of these works present relevant findings and valuable insights into the physical properties of CB doughs, it is difficult to translate their characterization methods into devices that can be installed and used in industrial production. Indeed, rheological and thermogravimetric measurements call for specific apparatus and potentially destructive analysis, and cryogenic spectroscopy is not feasible in an industrial scenario such as the Carasau baking industry. Among the presented techniques, dielectric spectroscopy (DS) is the most promising. DS is a non-destructive, powerful method for characterizing food materials [20,21,22]. By measuring, modeling and analyzing the dielectric signature (ϵ) of food materials as a function of frequency (f), it is possible to retrieve knowledge about microstructure and organoleptic properties. However, working in the low-frequency or radiofrequency range, as was done in ref. [19], may result in low specificity and poor accuracy if the measurements are performed at room temperature. Therefore, working at higher f, in the microwave regime (MW: 300 MHz–30 GHz), would ensure a better and deeper understanding of food material properties. Indeed, MW DS techniques have been used for determining apple maturity [20] or pork meat quality [21]. In addition, the development and dynamics of the apple-candying process, as well as its quality, has been monitored with MW DS [22]. When MW DS is used to preliminarily characterize a food product, novel applications can be developed. For instance, MW systems, such as antenna arrays to perform microwave imaging, have recently been gaining attention as tools for the detection of physical contamination in the food industry [23]. In ref. [24], it was shown that planar sensors could be used to assess the composition of vegetable oils, and in ref. [25], the authors demonstrated that food pathogens could be detected through variation in dielectric properties via impedance sensors. However, although MW DS is beginning to be adopted in the food industry, there is lack of understanding of MW dielectric properties of bread or dough, especially Carasau bread. In fact, in ref. [26], an open-ended coaxial line was used to measure the complex permittivity of commercial bread dough, in the range f∈[0.6,2.45] GHz, but without modeling and considering the effect of composition and different ingredients amounts. In ref. [27], indirect measurements, up to 6 GHz, were made of dielectric permittivity of dough with variable water and salt amount. No fitting or modeling was performed in this case. On the other hand, in ref. [28], the complex dielectric permittivity of white bread was measured from 0.1 to 1.8 GHz and modeled through mixing equations and polynomial fitting. None of the aforementioned works [26,27,28] dealt with the characterization of Carasau doughs, and the findings could not be transposed to this peculiar food product. Recently, in ref. [29], an MW DS study, up to 8.5 GHz, was carried out on different samples of Carasau dough, prepared with different semolina batches. In ref. [20], the best MW DS model was selected, and the variation in the dielectric permittivity was investigated using a third-order Cole–Cole model. The focus was on the change in dielectric properties during leavening. Table 1 summarizes the state of the art of MW DS of bread and dough. Table 1 shows that the maximum frequency used for characterization is f=8.5 GHz in ref. [29]. Furthermore, the DS data have not always been modeled, i.e., they have not been framed in a quantitative scheme. Combining this information with the fact that the effects that composition on the bread dough properties have not been considered, except in ref. [27], it is necessary to investigate and model how the composition of CB doughs affects their dielectric properties and model the MW response suitably.

Therefore, in this work, we aimed to perform an extensive and accurate microwave dielectric spectroscopy of Carasau bread doughs prepared with variable composition and to model their dielectric spectra. The effects of water, yeast and salt concentrations on the complex dielectric permittivity of the CB doughs was studied. Thorough modeling was carried out by relying on a third-order Cole–Cole model, to which the different dielectric spectra were fitted. Moreover, thermogravimetric analysis was performed to extract relevant figures of merit to derive the contribution of bound and free water content. The data on water content were preliminarily correlated to the dielectric response of the different CB doughs. The proposed analysis and findings are the first steps for designing an MW device to empower the CB industry.

## 2. Materials and Methods

In this work, we aimed to investigate the effects of CB dough composition on the dielectric spectrum at MW frequencies. To this aim, different dough samples were prepared by varying the nominal recipe. In particular, thanks to the collaboration with our industrial stakeholders, who have long-term experience and a deep knowledge of their product, we have learned that in the preparation of the dough the quantity of ingredients can be slightly different from the quantity required by the nominal recipe. In fact, it may happen that operators forget to add salt or yeast to the dough or that they adjust the recipe by adding more water, salt or yeast than the nominal recipe. The continuous involvement of industrial experts in this research has made it possible to outline the range of variations of these ingredients by defining a minimum and maximum value between which the nominal value lies. For each of the ingredients indicated, i.e., water, salt and yeast, the extremes of the range variation and the relative quantity indicated by the nominal recipe were selected for this first study. Carasau bread doughs with different quantities of ingredients were prepared and analyzed, for a total of seven samples types. We then characterized the different dough samples by performing MW DS measurements that, subsequently, were framed in a quantitative model. Furthermore, we performed thermogravimetric analysis to conduct a correlation analysis to better elucidate how the composition and microstructure relate to the dielectric properties.

### 2.1. Carasau Dough Preparation

The CB dough samples were prepared by using the following ingredients: commercial semolina (its basic chemical parameters are reported in Table 2 and Table 3); distilled water; commercial fresh brewer’s yeast (*Saccharomyces cerevisiae*, Lievital, IT); and commercial sea salt (Selex, IT). Assuming the semolina as the main component, with respect to its weight, we define W as the relative percentage water content, Y as the relative percentage yeast weight and NaCl as the relative percentage of salt weight.

In this work, a single batch of semolina wheat was used to prepare all samples. The semolina presents the same characteristics as the one tested in ref. [15] regarding its composition (carbohydrates, proteins, gluten, and fats) and gluten index, these values are reported in Table 3.

The dough preparation was performed by using a Sana Smart Breadmaker (SANABMS, Sana S.r.o., CZR) machine for dough kneading. For the investigations, the initial and nominal recipe was prepared using 300 g of semolina, 150 g of distilled water, 4.5 g of NaCl and 4.5 g of yeast. The composition in terms of weight percentage is reported in Table 2. Each sample was mixed for a fixed time before starting the measurement (20 min) at the fixed velocity of 88 rpm and at room temperature, as done in refs. [15,29]. These long mixing times were chosen to study the effect of overmixing on the dough properties and to understand how the addition of the different ingredients influences this phenomenon. The CB dough samples prepared with these ingredients and mixing times are called “B7ST20”. In this work, the effect of mixing time on the dielectric properties was not considered, and this processing parameter was kept constant.

As previously explained, it is common that some of the ingredients, such as water, salt and yeast, are added to the dough in different quantities than those indicated in the nominal recipe. In fact, it may happen that operators adjust the quantities of these ingredients, with respect to the nominal recipe, or forget to add salt or yeast during the production of Carasau bread (i.e., 0% of these elements in the dough). Thanks to the close collaboration with our industrial stakeholders, we have identified the minimum and maximum quantities that, for each ingredient, can be added to the dough during production. In other words, we have mapped the upper and lower limits for the weight fraction of water, yeast and salt. The ranges in variation found for these ingredients are as follows: water 46–54%, salt 0–2.5% and yeast 0–2.5%, as a weight percentage with respect to the quantity of semolina indicated in the nominal recipe (i.e., 300 g). For each ingredient, the minimum, maximum and nominal values were used for the preparation of dough samples to be analyzed. Therefore, regarding the amount of water, samples with W of 46%, 50%, and 54% weight fractions were prepared to investigate the contribution on the physical properties of CB dough. As regards the salt influence, the tested samples were composed considering NaCl=0%,1.5%,2.5%, always based on the semolina weight. For studying the yeast impact, we selected 0%,1.5% and 2.5%, as the Y weight fraction (still based on the semolina weight). The composition of all samples is reported in Table 2 with the respective nomenclature used to indicate each sample. The number of sampling points for each ingredient interval could be increased to perform a more in-depth analysis of the trends. However, considering that this work deals for the first time with the influence of the Carasau bread dough composition on the microwave dielectric properties, we believed that the study can initially be performed based on only three points. This allows, in this first analysis, to explore how much the dielectric spectra can vary as the ingredients vary within the relative ranges of variation and to approximately identify their trend. Moreover, it allows us to compare the dielectric spectra of the dough obtained with the nominal recipe with the dielectric spectra relating to the mixtures obtained with the maximum variations of these ingredients detectable in the production of Carasau bread. All analyses were performed considering the intervals given in Table 2. The following analyses were therefore performed on seven different CB dough samples and, for each of them, three kneading times were applied to replicate the sample, for a total of 21 experimental conditions. The results are presented as averages of each case.

### 2.2. Microwave Spectroscopy Characterization

The response of a material to the EM field in the MW range is expressed in terms of its complex permittivity $\epsilon =\epsilon^{\prime }-j\epsilon^{\prime \prime }$, where $\epsilon^{\prime }$ is the real part, which measures the electric energy stored in the material under test (MUT), and $\epsilon^{\prime \prime }$ is the imaginary part of the permittivity, which is related to the energy dissipated inside the MUT. Through MW broadband spectroscopy, it is possible to observe nano-scale dynamic relaxation and polarization mechanisms, thus accessing unique insights into the MUT properties.

**Figure 2:**
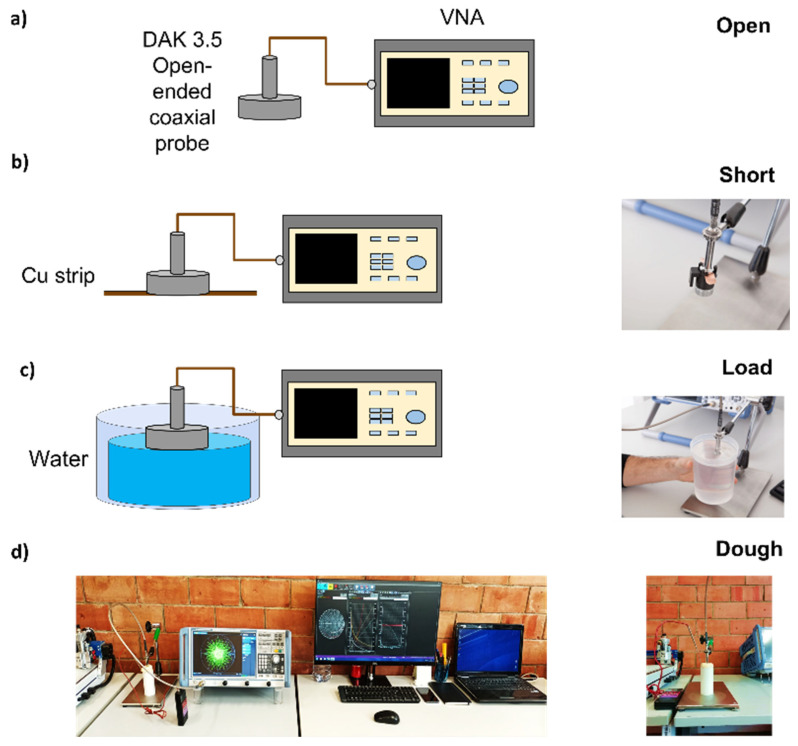
Calibration procedure: (**a**) open measurement, (**b**) short measurement and (**c**) load measurement (with a strip of copper, Cu), with 1 L de-ionized water. (**d**) Microwave spectroscopy measurement setup for the Carasau dough.

Considering the approach adopted in the literature (Table 1), in this work, the measurements of ϵ were carried out by using an open-ended coaxial probe (OCP) dielectric assessment kit (DAK) system, shown in Figure 2. To derive ϵ from the complex reflection coefficient at the probe–material interfaces, the OCP requires the open-short-load (OSL) calibration procedure. First, an open measurement is performed (Figure 2a), and the capacitance in air, due to the fringe field, is derived. A copper strip is used to short the outer and inner conductors of the probe, as shown in Figure 2b. Finally, a 1 L de-ionized water volume was used as reference load (Figure 2c) and its temperature was monitored using a digital PT100 thermometer (±0.05 °C accuracy). Using admittance or capacitive models, it is then possible to measure the dielectric properties of any materials [30]. The measurement system consisted of a vector network analyzer (VNA), Rhode & Schwarz ZNB 8 (9 KHz–8.5 GHz) and a 3.5 DAK-probe (SPEAG; www.speag.com, accessed on 20 April 2023), as shown in Figure 2d. The probe is connected to the VNA using a rigid, low-loss coaxial cable, and a lab jack is used to move the MUT toward the probe, as shown in Figure 2d. To the measured data, the combined variance, considered as sum of drift, random and systematic contribution (i.e., $s_{comb}=s_{drift}+s_{random}+s_{syst}$), at every frequency point, was considered, for a coverage factor k=2 for a 95% confidence interval [29].

### 2.3. Microwave Spectroscopy Modeling

Given the possibility in this work to measure the complex dielectric permittivity in a way different from the references reported in Table 1, we modeled the MW spectra of Carasau dough to investigate how the curves and the model parameters are affected by the pastry composition. Broadly speaking, ϵ varies with the working frequency f, i.e., ϵ(f). However, for food materials, the dielectric properties in the MW regime depend upon the composition, especially on the water content [19,20,21,22]. Therefore, more in general, we expect $\epsilon \left(f,W,Y,NaCl\right)$. In this work, we studied the spectra acquired by varying these compositional parameters. Recently, in ref. [29], it was shown that a non-resonant model, accounting for a distribution of relaxation times, could explain the MW spectrum of the Carasau dough prepared with the nominal recipe. Therefore, in this work we used the same model and expanded it to quantitatively understand the response of this food material by varying its water, salt and yeast content. In other words, we aimed at retrieving the coefficient of a given formula for all spectra of the seven samples reported in Table 2. Therefore, the dielectric permittivity was modeled as follows:

(1) $$\epsilon \left(\omega \right)=\epsilon_{\infty }+\sum_{q=1}^{N_{p}}\frac{∆\epsilon_{q}}{1+{\left(j\omega \tau_{q}\right)}^{1-\gamma_{q}}}+\frac{\sigma_{dc}}{j\omega \epsilon_{0}}$$

where ω=2πf is the angular frequency, $\epsilon_{\infty }$ is the dielectric permittivity at optical frequencies, and $N_{p}=3$ is the pole number. On the other hand, ∆ϵ is the difference between the static permittivity ($\epsilon_{s}$) and the permittivity at very high frequencies, i.e.,

(2) $$\Delta \epsilon_{q}=\epsilon_{s,q}-\epsilon_{\infty }$$

In Equation (1), $\tau_{q}$ is the *q*th relaxation time (in s), while γ is the so-called broadening or shape parameter, and $\sigma_{dc}$ and $\epsilon_{0}$ are the electrical conductivity (in S/m) and the vacuum dielectric permittivity, equal to 8.85 × 10^−12^ F·m^−1^, respectively.

Since ϵ(W,Y,NaCl), from Equation (1), we expect all parameters to be a function of water, yeast and salt content. To retrieve the $3\cdot N_{p}+2=11$ parameters of the proposed model, for each ith frequency point, we fitted the experimental data ($\epsilon_{m}$) measured using the protocol depicted in Figure 2 by minimizing the following cost function g, i.e.,

(3) $$g\left(x\right)={\left\{\frac{1}{N_{f}}\sum_{i=1}^{N_{f}}\left[{\left|\frac{\epsilon_{m,i}^{\prime }-\epsilon_{th,i^{\prime }}}{\epsilon_{m,i}^{\prime }}\right|}^{2}+{\left|\frac{\epsilon_{m,i}^{\prime \prime }-\epsilon_{th,i}^{\prime \prime }}{\epsilon_{m,i}^{\prime \prime }}\right|}^{2}\right]\right\}}^{1/2}$$

In Equation (3), x is the vector of unknown parameters, while $N_{f}$ is the number of frequency points, and $\epsilon_{th}$ is the theoretical permittivity evaluated with Equation (1). To minimize Equation (3), we used the genetic algorithm routine described and used in ref. [29]. Briefly, the initial population was set to $3\cdot 10^{4}$ individuals, using maximum 500 iterations, by imposing crossover and mutation probabilities equal to 0.9 and 0.1, respectively. To find the best x that minimizes Equation (3), for the 11 parameters of Equation (1), the solution space is provided in Table 4.

### 2.4. Thermogravimetric Characterization

For each CB dough sample from Table 2, a small quantity (about 100 μg) of dough (prepared just before) was put into an alumina crucible and inserted into a thermogravimetric (TGA) device (TA Instruments, New Castle, DE, USA, SDT-Q600). Then, the sample was heated up to a maximum temperature $T_{max}=600$ °C with a temperature ramp $\frac{\Delta T}{\Delta t}=5$ °C/min. For each run, the weight loss of the sample was registered. Furthermore, the percentage reduction $\left(W_{\%}(T)\right)$ and the first derivative of the latter with respect to the temperature (i.e., $\frac{\partial W_{\%}}{\partial T}$) were calculated. Finally, the experimental data obtained for each sample were elaborated to derive the (i) total, (ii) free and (iii) bound water content. Two replicate measurements for every sample were performed, and then the average value was taken as a result.

### 2.5. Correlation Analysis

The results of the thermogravimetric determinations were used for attempting to establish correlations between the total, free and bound water content and the dielectric parameters of the third-order Cole–Cole model.

Recalling that W is the weight fraction of the water added to the dough with respect to the semolina weight, we defined $W_{tot}$ as the estimated total weight fraction of water in the dough, while $W_{f}$ is the free water weight fraction in the dough and $W_{b}$ is the weight fraction of water that is in a bound state in the dough. It must be noted that $W_{tot}=W_{f}+W_{b}$. These terms were estimated through the thermogravimetric analyses, as stated before.

The following relationships between the water content in the dough and the dielectric model parameters were hypothesized:

(4) $$\begin{array}{l} W_{f}=b_{1,1}\cdot \epsilon_{\infty }+b_{1,2}\cdot ∆\epsilon_{1}+b_{1,3}\cdot \tau_{1}+b_{1,4}\cdot \gamma_{1}+b_{1,5}\cdot ∆\epsilon_{2}\\ +b_{1,6}\cdot \tau_{2}+b_{1,7}\cdot \gamma_{2}+b_{1,8}\cdot ∆\epsilon_{3}+b_{1,9}\cdot \tau_{3}+b_{1,10}\cdot \gamma_{3}\\ +b_{1,11}\cdot \sigma_{dc}+b_{1,12} \end{array}$$

(5) $$\begin{array}{l} W_{b}=b_{2,1}\cdot \epsilon_{\infty }+b_{2,2}\cdot ∆\epsilon_{1}+b_{2,3}\cdot \tau_{1}+b_{2,4}\cdot \gamma_{1}+b_{2,5}\cdot ∆\epsilon_{2}\\ +b_{2,6}\cdot \tau_{2}+b_{2,7}\cdot \gamma_{2}+b_{2,8}\cdot ∆\epsilon_{3}+b_{2,9}\cdot \tau_{3}+b_{2,10}\cdot \gamma_{3}\\ +b_{2,11}\cdot \sigma_{dc}+b_{2,12} \end{array}$$

(6) $$\begin{array}{l} W_{tot}=b_{3,1}\cdot \epsilon_{\infty }+b_{3,2}\cdot ∆\epsilon_{1}+b_{3,3}\cdot \tau_{1}+b_{3,4}\cdot \gamma_{1}+b_{3,5}\cdot ∆\epsilon_{2}\\ +b_{3,6}\cdot \tau_{2}+b_{3,7}\cdot \gamma_{2}+b_{3,8}\cdot ∆\epsilon_{3}+b_{3,9}\cdot \tau_{3}+b_{3,10}\cdot \gamma_{3}\\ +b_{3,11}\cdot \sigma_{dc}+b_{3,12} \end{array}$$

The coefficients of these relationships were estimated through the polynomial least squares fitting method.

## 3. Results

### 3.1. Microwave Spectroscopy Investigation

**Figure 3:**
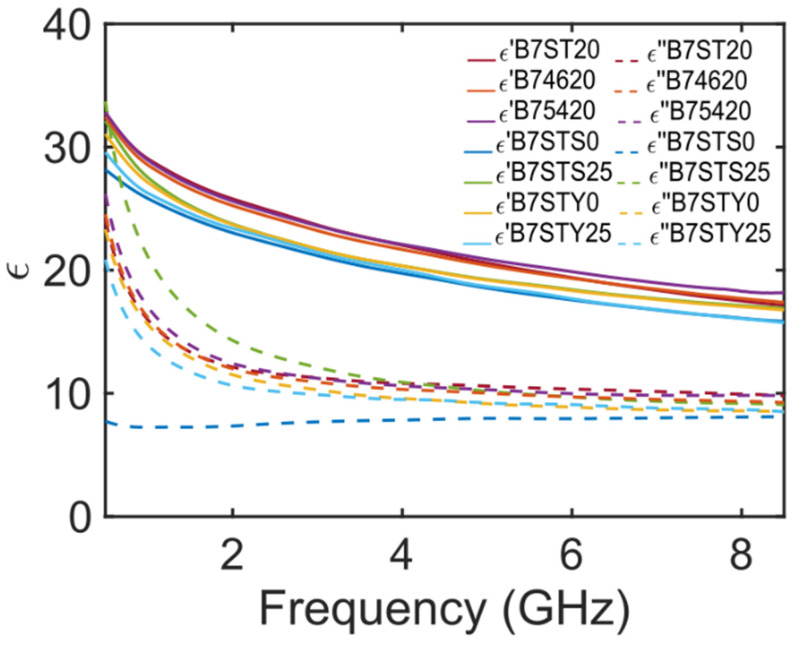
Complex dielectric permittivity (ϵ), in real ($\epsilon^{\prime }$) and imaginary ($\epsilon^{\prime \prime }$) parts, of seven different Carasau bread dough samples obtained varying the weight fraction of water, yeast and salt.

The dielectric spectra of the real ($\epsilon^{\prime }$) and imaginary ($\epsilon^{\prime \prime }$) parts of the complex dielectric permittivity of the seven different Carasau bread doughs are reported in Figure 3. It can be seen that as the water content W increases, the real part of the permittivity also increases (see B74620, B7ST20, B75420 curves), throughout the considered MW band. For a dough sample with Y=1.5%, NaCl=1.5%, the real part of the dielectric permittivity is higher than in the other four cases. Regarding the imaginary part of the dielectric permittivity, from Figure 3 it is evident that the MUT presents significant losses ($\epsilon^{\prime \prime }>20$ for f~0.5 GHz and $\epsilon^{\prime \prime }>10$ at 8.5 GHz), and the out-of-phase component of the permittivity increases as the water weight fraction increases. However, the highest losses were not observed for the wetter dough sample.

On the other hand, when no salt is used in the dough preparation (NaCl=0%), a drastic decrease in $\epsilon^{\prime }$ is observed (~15%) in the lower bound of the frequency (B7STS0 in Figure 3). The salt absence is of less relevance for higher frequencies. In fact, by reducing the ionic content in the dough, less free charges are available and the losses due to ionic currents decrease ($\epsilon^{\prime \prime }<10\forall f$). As the salt content in the CB dough increases to the maximum value of 2.5% (B7STS25 in Figure 3), the MW spectrum is significantly altered. Indeed, the increased presence of ions lead to a dielectric strength $\epsilon^{\prime }$ comparable to that of a dough made with W=54%, below 1 GHz. The most remarkable effect of salt addition on the dough MW response is the large increase in the $\epsilon^{\prime \prime }$ values, which rise to a maximum of 35 at 0.5 GHz and are very large ($\epsilon^{\prime \prime }$~13.5) up to 4 GHz. After this frequency, the dielectric loss of the dough tends to decrease and is very similar to other samples made with a different compositions.

Regarding the yeast weight fraction and its effects on the CB dough dielectric properties, Figure 3 (B7STY0) shows that the absence of a leavening agent reduces the real part of the dielectric permittivity. The $\epsilon^{\prime }$ values for a dough kneaded without yeast are very similar to that measured for a very salty dough. In fact, for frequencies above 2 GHz, the curves are almost indistinguishable, considering the accuracy and precision of the DAK OCP. These considerations are true for the real part of the dielectric permittivity, but for $\epsilon^{\prime \prime }$, in Figure 3, it is possible to observe that the curve is very similar to those of samples B7ST20 or B74620. On the other hand, for a dough sample loaded with the highest weight fraction of yeast (B7STY25-Y=2.5%), for $\epsilon^{\prime }$ there is a noticeable overlap with the MW response of sample B7STS0 (NaCl=0%). In other words, a dough prepared with a large yeast concentration at MW has a real part of the dielectric permittivity very similar to a dough prepared without salt addiction. Regarding the imaginary part of the dielectric permittivity of the sample B7STY25, it can be observed that it is higher than the losses of the sample B7STS0, but generally lower than the other curves for frequencies up to 4 GHz, where $\epsilon^{\prime \prime }$ tends to be almost identical to B7STY0.

Figure 3 indicates that slight modifications in the composition of Carasau dough samples can lead to significant variation in the dielectric permittivity in the MW regime. Furthermore, we can infer that the largest contrast between the dielectric properties of different dough samples occurs below 4 GHz.

### 3.2. Modeling of the MW Dielectric Properties

The MW DS data were fitted to the proposed Cole–Cole model using the genetic algorithm and the fitting function described previously. The fitting errors on the real and imaginary parts are reported in Table 5.

The variation in the model parameters as a function of the percentage of water, salt and yeast was studied. For each of these parameters, trend in the values were observed.

**Figure 4:**
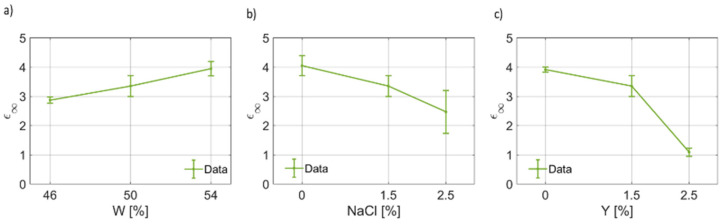
(**a**) Trend of the dielectric permittivity at very high frequency ($\epsilon_{\infty })$ as a function of the bread dough water weight fraction ($\epsilon_{\infty }(W)$). (**b**) Trend of the dielectric permittivity at very high frequency as a function of the salt weight fraction ($\epsilon_{\infty }(NaCl)$). (**c**) Trend of the dielectric permittivity at very high frequency as a function of the yeast weight fraction ($\epsilon_{\infty }(Y)$).

**Figure 5:**
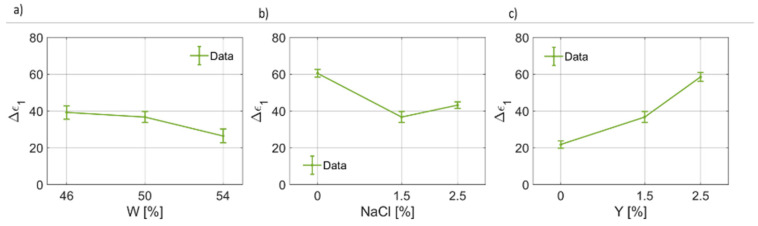
(**a**) Trend of the first pole static permittivity (${\Delta \epsilon }_{1})$ as a function of the bread dough water content (${\Delta \epsilon }_{1}(W)$). (**b**) Trend of the first pole static permittivity as a function of the salt concentration (${\Delta \epsilon }_{1}(NaCl)$). (**c**) Trend of the first pole static permittivity as a function of the yeast concentration (${\Delta \epsilon }_{1}(Y)$).

**Figure 6:**
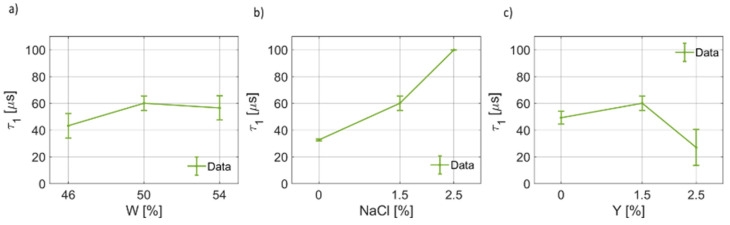
(**a**) Trend of the first relaxation time $\left(\tau_{1}\right)$ as a function of the bread dough water content ($\tau_{1}(W)$). (**b**) Trend of the first relaxation time as a function of the salt concentration ($\tau_{1}(NaCl)$). (**c**) Trend of the first relaxation time as a function of the yeast concentration ($\tau_{1}(Y)$).

**Figure 7:**
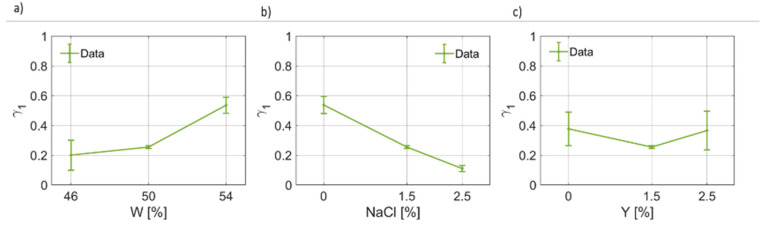
(**a**) Trend of the first broadening parameter ($\gamma_{1})$ as a function of the bread dough water content ($\gamma_{1}(W)$). (**b**) Trend of the first broadening parameter as a function of the salt concentration ($\gamma_{1}(NaCl)$). (**c**) Trend of the first broadening parameter as a function of the yeast concentration ($\gamma_{1}(Y)$).

In Figure 4, the results of the fitting for the dielectric permittivity at optical frequency are reported. It can be noticed that $\epsilon_{\infty }(W)$ increases from ~3 to ~4 units (Figure 4a). On the other hand, for $\epsilon_{\infty }(NaCl)$, as the salt weight fraction increases, the dielectric permittivity at high frequencies decreases linearly up to a minimum value of ~2.5, on average. Similarly, for increasing yeast concentration in the dough, $\epsilon_{\infty }$ decreases (Figure 4c). In Figure 5, the retrieved values for the first pole ${\Delta \epsilon }_{1}$ have been provided as a function of water, salt and yeast weight fraction. Figure 5a shows that the static dielectric permittivity of the first pole decreases as the water content increases, with a linear trend, for the range and points considered in this work. Figure 5b indicates that the static dielectric permittivity of the first pole depends on the salt concentration, but generally lowers as the salt concentration increases. This result is coherent with the curves given in Figure 4 for B7STS0 and B7STS25. Regarding ${\Delta \epsilon }_{1}(Y)$, from Figure 5c, it can be noticed that the static coefficient of the first pole increases as the yeast concentration increases. In Figure 6, the variation in the first relaxation time $\tau_{1}$ for the three ingredients and components are reported. This relaxation time is on the order of tens to hundreds of μs. As can be seen from Figure 6a, the relaxation time is not constant and depends on the water weight fraction. In Figure 6b, the trend $\tau_{1}(NaCl)$ can be considered to be a linear function. Indeed, the relaxation time almost doubles for NaCl=2.5%. The increased relaxation time is responsible for the increased losses and for the characteristics curves that are reported in Figure 3. Furthermore, a non-linear variation in $\tau_{1}$ with yeast concentration must be reported. A maximum value in the relaxation time is observed for the nominal recipe. In Figure 7, the coefficients for the first broadening parameter for the seven different samples are reported.

**Figure 8:**
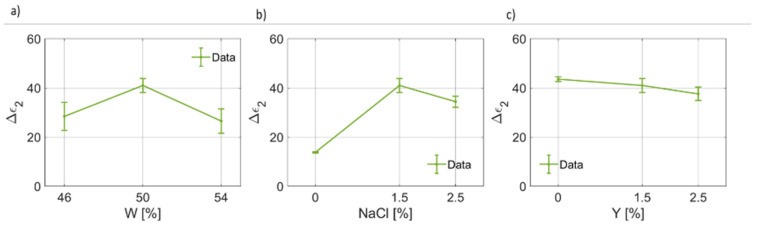
(**a**) Trend of the second pole static permittivity (${\Delta \epsilon }_{2})$ as a function of the bread dough water content (${\Delta \epsilon }_{2}(W)$). (**b**) Trend of the second pole static permittivity as a function of the salt concentration (${\Delta \epsilon }_{2}(NaCl)$). (**c**) Trend of the second pole static permittivity as a function of the yeast concentration (${\Delta \epsilon }_{2}(Y)$).

**Figure 9:**
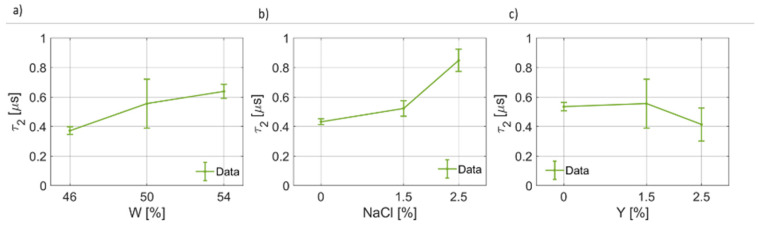
(**a**) Trend of the second relaxation time ($\tau_{2})$ as a function of the bread dough water content ($\tau_{2}(W)$). (**b**) Trend of the second relaxation time as a function of the salt concentration ($\tau_{2}(NaCl)$). (**c**) Trend of the second relaxation time as a function of the yeast concentration ($\tau_{2}(Y)$).

**Figure 10:**
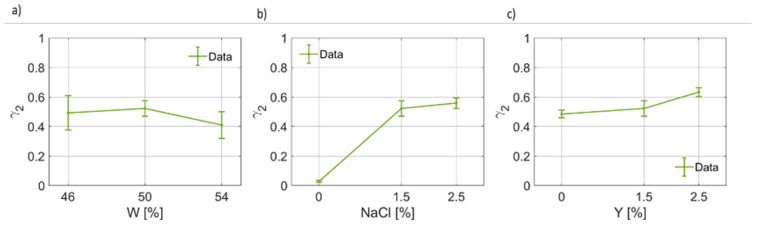
(**a**) Trend of the second broadening parameter ($\gamma_{2}$) as a function of the bread dough water content ($\gamma_{2}(W)$). (**b**) Trend of the second broadening parameter as a function of the salt concentration ($\gamma_{2}(NaCl)$). (**c**) Trend of the second broadening parameter as a function of the yeast concentration ($\gamma_{2}(Y)$).

The results for the coefficients of the second pole are given in Figure 8, Figure 9 and Figure 10. For the static permittivity of the second pole, an evident variation of ${\Delta \epsilon }_{2}(W)$ and ${\Delta \epsilon }_{2}(NaCl)$ was found (Figure 8a,b). The highest values for ${\Delta \epsilon }_{2}$ were found for the nominal case, while reductions are observed in all other cases. It is worth highlighting that a linear, but slight, reduction in ${\Delta \epsilon }_{2}$ as a function of Y were identified. On the other hand, for the second relaxation time, which is on average about an order of magnitude lower than the first one ($\tau_{1}>\tau_{2}$), the fitting coefficients are reported in Figure 9. $\tau_{2}$ as a function of water content and salt increases as W and NaCl increases, apparently in a linear fashion and in a non-linear way, respectively (Figure 9a,b). On the other hand, $\tau_{2}$ is poorly affected by the yeast concentration and, as can be seen from Figure 9c, its value decreases significantly only for Y=2.5%. In Figure 10, the results for the second broadening parameter $\gamma_{2}$ are given. It can be noticed that $\gamma_{2}(W)$ has a narrow quadratic distribution around ~0.49, whereas $\gamma_{2}(NaCl$) increases nonlinearly, thus explaining the behavior of the curves reported in Figure 3, and $\gamma_{2}\left(Y\right)$ slightly increases as the yeast fraction in the dough increases.

**Figure 11:**
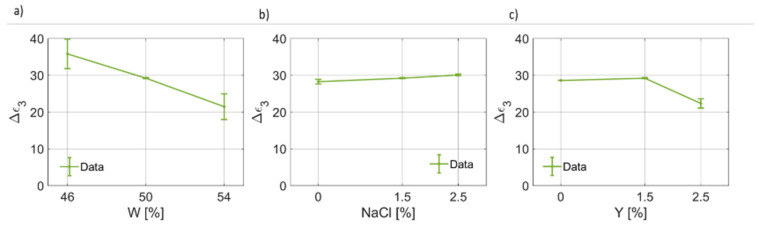
(**a**) Trend of the third pole static permittivity (${\Delta \epsilon }_{3}$) as a function of the bread dough water content (${\Delta \epsilon }_{3}(W)$). (**b**) Trend of the third pole static permittivity as a function of the salt concentration (${\Delta \epsilon }_{3}(NaCl)$). (**c**) Trend of the third pole static permittivity as a function of the yeast concentration (${\Delta \epsilon }_{3}(Y)$).

**Figure 12:**
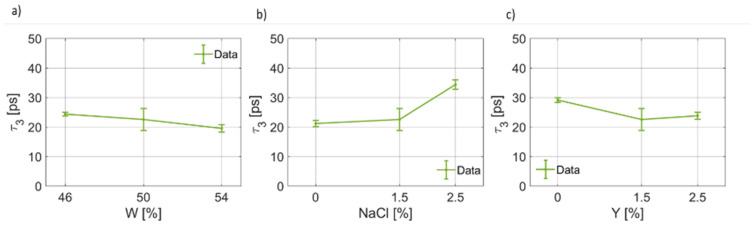
(**a**) Trend of the third relaxation time ($\tau_{3}$) as a function of the bread dough water content ($\tau_{3}(W)$). (**b**) Trend of the third relaxation time as a function of the salt concentration ($\tau_{3}(NaCl)$). (**c**) Trend of the third relaxation time as a function of the yeast concentration ($\tau_{3}(Y)$).

**Figure 13:**
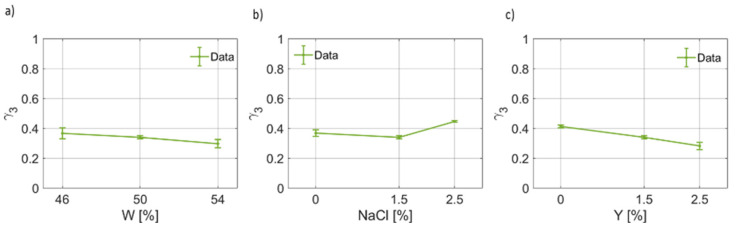
(**a**) Trend of the third broadening parameter ($\gamma_{3}$) as a function of the bread dough water content ($\gamma_{3}(W)$). (**b**) Trend of the third broadening parameter as a function of the salt concentration ($\gamma_{3}(NaCl)$). (**c**) Trend of the third broadening parameter as a function of the yeast concentration ($\gamma_{3}(Y)$).

As regards the third pole of the proposed Cole–Cole model, the coefficients are reported in Figure 11, Figure 12 and Figure 13. By observing Figure 11, it is possible to notice that for the third pole of the proposed Cole–Cole model, $\Delta \epsilon_{3}<\Delta \epsilon_{1}$. In Figure 11a, a decreasing trend of ${\Delta \epsilon }_{3}\left(W\right)$ has been found. On the other hand, the static permittivity of the third pole increases as the salt weight fraction increases (Figure 11b), whereas a reduction is observed as a function of the yeast concentration. In Figure 12, the behavior of the relaxation time $\tau_{3}$, which is much lower than the others and is on the order of tens of ps, as a function of the water, salt and yeast weight fraction is reported. It can be noticed that, as the water fraction increases, the relaxation time decreases. The salt concentration, instead, has a more marked effect on the increase in the relaxation time, which is a function of NaCl. In other words, as a larger ion concentration is present in the dough, the larger the relaxation time becomes. In a different way, as the weight fraction of the leavening agent increases, a lower time is required to the relaxation process to be complete for this third contribution to the overall dielectric response, i.e., $\tau_{3}(Y)$ decreases.

In Figure 13, the values derived for the fitting parameter $\gamma_{3}$ as a function of water, salt and yeast concentration are reported. The third broadening parameter is almost constant and not so sensitive to the water content and yeast fraction, as can be noticed in Figure 13a,c, for which a slight decrease is observed. In Figure 13b, a slight increase in the broadening parameter for increasing salt concentration is reported.

**Figure 14:**
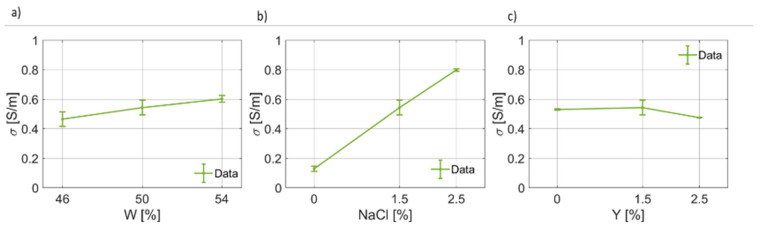
(**a**) Trend of the static electric conductivity ($\sigma_{dc}$) as a function of the bread dough water content ($\sigma_{dc}(W)$). (**b**) Trend of the static electric conductivity as a function of the salt concentration ($\sigma_{dc}(NaCl)$). (**c**) Trend of the static electric conductivity as a function of the yeast concentration ($\sigma_{dc}(Y)$).

Finally, the values found for the static electric conductivity, $\sigma_{DC}$, are reported in Figure 14. As reasonably expected, if more water is available in the dough matrix, $\sigma_{DC}$ is higher (Figure 14a). Similarly, $\sigma_{DC}(NaCl)$ is expected to increase as the salt weight fraction increases, and this was verified, as shown in Figure 14b. As expected, the yeast fraction does not influence the value of the static dielectric conductivity, poorly contributing to the charge movement in the dough system.

Considering the very low errors reported in Table 5, the behavior of the eleven coefficients for the seven Carasau bread dough samples is valid, effective and trustworthy. However, the information derived from the MW DS analysis must be complemented and supported by other physical characterization methods to provide more insight into the microstructure of the food material. Therefore, in the following, the findings about the thermogravimetric analysis and the correlation study are presented.

### 3.3. Thermogravimetric Analysis

**Figure 15:**
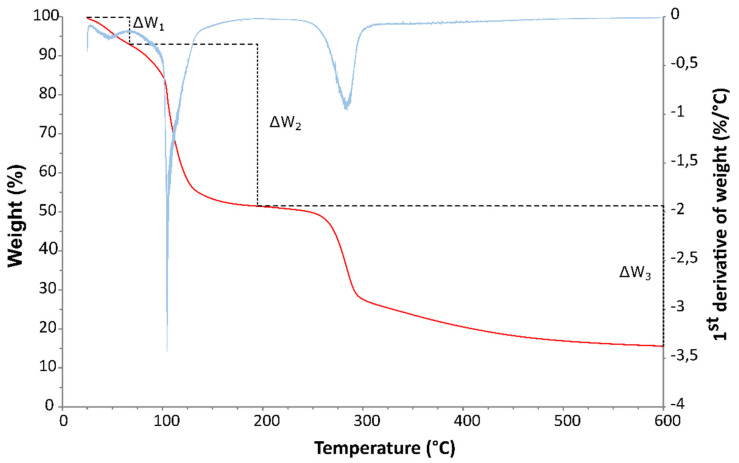
Percent weight loss (red line) and its first derivative with respect to the temperature (blue line) for a dough sample. $∆W_{1}$ represents the free water loss and $∆W_{2}$ indicates the bound water loss.

The thermogravimetric analysis data were elaborated to obtain the free, bound and total water content following the methodology already used by other authors [31,32], as shown in Figure 15. According to this methodology, the free water corresponds to the weight loss between the starting point and the local maximum of the first derivative just before the water evaporation peak (which can be found around 100 °C), and the bound water corresponds to the weight loss between the latter maximum and the maximum immediately following the water evaporation peak. In Figure 15, they are indicated with $∆W_{1}$ and $∆W_{2}$, respectively.

### 3.4. Correlation Analysis

The results of the correlation analysis for Equations (4)–(6) are reported in Table 6. In general, for W∈[46, 54]%, with respect to the semolina weight, an increase in water quantity tends to increase the bounded water fraction, while poorly affecting the free water concentration.

Regarding the free water in the dough, by observing Table 6, it is possible to notice that it is negatively correlated with $\epsilon_{\infty }$, positively correlated with $\Delta \epsilon_{1}$, $\tau_{1}$ and $\gamma_{1}$, and also with the parameters of the second pole ($\Delta \epsilon_{2}$, $\tau_{2}$), but not with $\gamma_{2}$. $W_{f}$ is negatively correlated with $\Delta \epsilon_{3}$, but it is positively related to $\tau_{3}$ and $\gamma_{3}$. Finally, the higher the electrical conductivity values, the higher the total water in the dough. These results are interesting and already similar to the trends and findings from Figure 3, Figure 4, Figure 5, Figure 6, Figure 7, Figure 8, Figure 9, Figure 10, Figure 11, Figure 12, Figure 13 and Figure 14.

However, when the contributions of bound water to the dielectric response are analyzed, more interesting features are found. In particular, the content of bound water ($W_{b}$) is more evident if parameters such as $\gamma_{2}$ and $\sigma_{dc}$ are considered. In fact, a negative correlation coefficient between $W_{b}$ and $\sigma_{dc}$ was found.

As regards the total water, a significantly different correlation coefficient with $\tau_{1},\gamma_{1},{\Delta \epsilon }_{2},\tau_{2}$ was obtained.

## 4. Conclusions

Carasau bread is a traditional flat bread produced in Sardinia. The small-scale industries which automated the manufacturing process of this food product would like to further advance the quality, while reducing wastage and controlling the production at different levels. In this framework, microwave spectroscopy can provide a unique, non-destructive and cost-effective opportunity to assess the dough characteristics and properties for empowering the productive process. Therefore, this work dealt with the investigation of the complex permittivity spectra of Carasau bread doughs, at microwave frequencies (up to 8.5 GHz), considering the influence of composition, i.e., the relative amount of water, salt and yeast. Measurements were performed using an open-ended coaxial probe. The obtained microwave spectra highlight that the real part of the complex permittivity is slightly affected by the dough composition, whilst a large variation in the imaginary part of the complex dielectric permittivity can be observed. In particular, these variations in the dielectric signature occur in the frequency range below 4 GHz. To model the obtained spectra, the experimental data were successfully fitted to a third-order Cole–Cole model, with a maximum error of 1.58% for the real part and 1.60% for the imaginary parts of permittivity. The parameters for all the investigated samples were derived and their variations with respect to water, salt and yeast content were also studied. Acorrelation analysis of microwave data with thermogravimetric data was performed. This last analysis highlighted that an increase in water quantity tends to increase the bounded water fraction, whereas it affects the concentration of free water only marginally.

Future works may deal with the investigation of new parts of the microwave spectrum (f>10 GHz) for designing, studying and developing innovative devices to be used as instruments and tools for product quality assessment. Future analyses will deal with the quantification of the variation of the dielectric properties per percentage of water, yeast and salt using more sampling points for the composition interval. The methodology and findings of this study could be useful for beginning the production of gluten-free Carasau bread [33], or for innovating the production using plant proteins [34].

## Figures and Tables

**Table 1:** State of the art analysis of microwave dielectric spectroscopy of bread and dough.

Frequency Range (GHz)	Measurement Method	Modeling (Yes/No)	Composition Effects (Yes/No)	Ref.
0.6–2.45	Open-ended coaxial line	No	No	[26]
0.3–6	Coaxial cell	No	Yes	[27]
0.1–1.8	OCP ^1^	Yes (Mixing eqs., Polynomial fitting)	No	[28]
0.5–8.5	OCP	Yes (Mixing eqs., Debye, Cole–Cole fitting)	No	[29]
0.5–8.5	OCP	Yes (Cole–Cole fitting)	Yes	This work

^1^ OCP = Open-ended coaxial probe.

**Table 2:** Compositions and nomenclatures of the different Carasau bread dough samples. The nominal Carasau dough is B7ST20. B7 refers to the batch of semolina used, ST refers to standard dough and 20 refers to 20 min of kneading. For the water content variation (*W*) ST is replaced with the relative water weight percentage. For yeast (*Y*) and salt (*NaCl*) variation, 20 is replaced with ‘Y’ or ‘NaCl’ followed by the relative ingredient weight percentage.

Sample Name	Water (W, %)	Yeast (Y, %)	Salt (NaCl, %)
B7ST20	50	1.5	1.5
B74620	46	1.5	1.5
B75420	54	1.5	1.5
B7STS0	50	1.5	0
B7STS25	50	1.5	2.5
B7STY0	50	0	1.5
B7STY25	50	2.5	1.5

**Table 3:** Chemical parameters of the commercial semolina batch used in this study [15].

Element	%
Carbohydrates	71
Fats	1.5
Proteins	11.7
Gluten	8.7
Gluten index	88.00

**Table 4:** Parameter space for the genetic algorithm fitting routine. $\epsilon_{s}$ refers to the static permittivity.

Parameter	Min.	Max.	Definition
$\epsilon_{\infty }$	1	15	Dielectric permittivity at optical frequencies
∆ϵ	1	80	$∆\epsilon =\epsilon_{s}-\epsilon_{\infty }$
τ (s)	10^−13^	10^−3^	Relaxation time
γ	0	1	Broadening parameter
$\sigma_{dc}$ (S/m)	10^−6^	2	Static electric conductivity

**Table 5:** Fitting errors on both the real and imaginary parts for the seven Carasau bread samples. The nominal Carasau dough is B7ST20. B7 refers to the batch of semolina used, ST refers to standard dough, and 20 refers to 20 min of kneading. For the water content variation (*W*) ST is replaced with the relative water weight percentage. For yeast (*Y*) and salt (*NaCl*) variation, 20 is replaced with ‘Y’ or ‘NaCl’ followed by the relative ingredient weight percentage.

Sample Name	$\mathrm{Average}\;\mathrm{Relative}\;\mathrm{Percentage}\;\mathrm{Error}\;\mathrm{on}\;\epsilon^{\prime }$ (%)	$\mathrm{Average}\;\mathrm{Relative}\;\mathrm{Percentage}\;\mathrm{Error}\;\mathrm{on}\;\epsilon^{\prime \prime }$ (%)
B7ST20	0.89	0.62
B74620	0.53	0.53
B75420	1.58	1.28
B7STS0	1.38	1.60
B7STS25	1.39	0.90
B7STY0	1.16	0.95
B7STY25	0.52	0.86

**Table 6:** Values of the coefficients $b_{i,j}$ retrieved from the linear correlation analysis. $W_{f}$ refers to the free water weight fraction, $W_{b}$ refers to the bound water weight fraction, and $W_{tot}$ is the weight fraction of water used in the dough. $\epsilon_{\infty }$ is the dielectric permittivity at optical frequencies, ∆ϵ is the difference between the static permittivity ($\epsilon_{s}$) and the permittivity at very high frequencies, τ is the relaxation time, γ is the broadening parameter, and $\sigma_{dc}$ is the electrical conductivity.

	$W_{f}$	$W_{b}$	$W_{tot}$	
	$b_{1,j}$	$b_{2,j}$	$b_{3,j}$	
$b_{i,1}$	−9.65·10^−1^	−4.54	−5.71	$\epsilon_{\infty }$
$b_{i,2}$	1.84·10^−1^	−5.29·10^−1^	−3.56·10^−1^	$∆\epsilon_{1}$
$b_{i,3}$	1.34 s^−1^	5.31·10^−1^ s^−1^	2.184 s^−1^	$\tau_{1}$
$b_{i,4}$	10.05	4.48	14.61	$\gamma_{1}$
$b_{i,5}$	2.62·10^−1^	−5.18·10^−1^	−2.59·10^−1^	$∆\epsilon_{2}$
$b_{i,6}$	9.99·10^−1^ s^−1^	9.996·10^−1^ s^−1^	1.15 s^−1^	$\tau_{2}$
$b_{i,7}$	−24.71	40.27	15.59	$\gamma_{2}$
$b_{i,8}$	−6.65·10^−1^	−1.112	−1.838	$∆\epsilon_{3}$
$b_{i,9}$	1.00 s^−1^	1.00 s^−1^	1.69 s^−1^	$\tau_{3}$
$b_{i,10}$	2.778	−42.39	−41.20	$\gamma_{3}$
$b_{i,11}$	12.78 S^−1^·m	−3.18·10^−1^ S^−1^·m	13.28 S^−1^·m	$\sigma_{dc}$ (S/m)
$b_{i,12}$	18.43	111.6	1332	-

## Data Availability

The data presented in this study are available on request from the corresponding author. The data are not publicly available due to information that could compromise ongoing research. Data is contained within the article. The data used to support the findings of this study can be made available by the corresponding author upon request.

## Abbreviations

CB	Carasau Bread
DAK	Dielectric Assessment Kit
DS	Dielectric Spectroscopy
FB	Flat Bread
MUT	Material Under Test
MW	Microwaves
NMR	Nuclear Magnetic Resonance
OCP	Open-ended Coaxial Probe
OSC	Open-Short-Load
TGA	Thermogravimetric Analysis
VNA	Vector Network Analyzer
WSN	Wireless Sensors Network
